# Immunocompetent Lung-on-a-Chip: Engineering the Pulmonary Immune Microenvironment for Disease Modeling

**DOI:** 10.3390/bios16100547

**Published:** 2026-09-30

**Authors:** Xingrui Li, Yinan Li, Jiashuang Xu, Rui Sun, Wenao Liu, Xiaofeng Chen, Lin Zhu, Qiongzhen Zhao, Jinyao Li, Yuan Li, Huimin Zhang, Weidong Huang

**Affiliations:** 1 School of Pharmaceutical Sciences & Institute of Materia Medica, College of Life Science and Technology, Xinjiang University, Urumqi 830046, China; xrli@xju.edu.cn (X.L.); 15754436918@163.com (Y.L.);; 2 School of Pharmaceutical Sciences, Xiamen University, Xiamen 361005, China; 3 Key Laboratory of Agro-Forestry Environmental Processes and Ecological Regulation of Hainan Province, School of Environmental Science and Engineering, Hainan University, Haikou 570228, China; 4 Jianyin Hospital, Urumqi 830046, China

**Keywords:** lung-on-a-chip, immune microenvironment, adaptive immunity, microfluidics

## Abstract

Lung-on-a-chip (LOC) models are increasingly used to study pulmonary disease, yet most platforms are still judged primarily by structural fidelity rather than immune function. Here, we review the emergence of immunocompetent LOCs and propose a functional grading framework to benchmark immune integration, from barrier-only models to innate immune surveillance, adaptive immune responses, and lymphoid structure reconstitution. Using this framework, we assess how engineering parameters, including materials, mechanics, fluidics, extracellular matrix, and immune cell sourcing, define the level of immunocompetence that a platform can achieve. We show that the field has progressed considerably in recapitulating innate immunity but remains stalled at the transition to antigen-specific T- and B-cell responses, because current platforms struggle to reconcile the prolonged cell–cell contacts required for adaptive priming with the continuous perfusion needed for tissue homeostasis. Overcoming this gap will demand longer-term culture stability, improved strategies for autologous multi-lineage cell sourcing, and the integration of LOCs with multi-organ immune circuits, spatial multi-omics, and AI-driven multimodal data analytics. Together, these advances are likely to define the next generation of translational pulmonary immune models.

## 1. Background

The lung is a continuously exposed immunological interface. Beyond its primary role in gas exchange, the pulmonary immune microenvironment orchestrates complex responses that range from effective pathogen clearance to tissue repair [1]. Dysregulation of this delicate immune balance is a central pathological feature in the progression of infectious diseases (e.g., COVID-19, tuberculosis), chronic inflammatory conditions, and lung cancer. In oncology, the clinical success of immune checkpoint inhibitors (ICIs) and chimeric antigen receptor T-cell (CAR-T) therapies has underscored that therapeutic efficacy is governed not merely by tumor genetics but also by the heterogeneity of the local immune microenvironment [2]. Therefore, the accurate recapitulation of these immune interactions in vitro is imperative for dissecting disease mechanisms and accelerating drug development [3,4].

Conventional models remain insufficient for this purpose, as summarized in Table 1, which evaluates representative platforms across key immunological capability dimensions. Each entry reflects commonly reported characteristics rather than fixed properties of a given model type. Two-dimensional cultures lack tissue architecture and multicellular immune crosstalk [5]. Three-dimensional cultures and organoids better preserve epithelial heterogeneity, but they often lack vasculature, circulating immune cells, stromal support, and physiological mechanical cues [6,7]. Similarly, 3D gel cultures are insufficient in capturing spatiotemporal dynamics of physiological mechanics and substance exchange [8,9]. This limitation is particularly evident in the context of physiological forces, which are critical for lung function [10]. Moreover, the absence of key stromal components in organoids often disrupts immune cell recruitment and function [11,12,13]. Animal models provide systemic context, yet species differences in immune development and activation limit their translational value [14,15], contributing to a high attrition rate in drug development. Approximately 89% of therapies effective in mice fail in human clinical trials due to lack of efficacy or unforeseen toxicity [16,17,18]. Together, these limitations create a persistent gap between preclinical findings and human pulmonary immunobiology.

LOC technology has emerged as a powerful alternative by leveraging microfluidics and tissue engineering to reconstruct the alveolar–capillary interface [19]. Early studies focused mainly on structural biomimicry, such as barrier architecture, air-liquid interface culture, and breathing-like mechanical strain [19,20,21,22]. More recent work has shifted toward immune integration by incorporating macrophages, neutrophils, monocytes, and, in some cases, lymphocytes into chip-based lung models [23,24]. This transition is critical because structural fidelity alone is not sufficient for modeling infection, chronic inflammation, fibrosis, or immunotherapy response. What matters is whether the engineered system supports immune functions such as recruitment, trafficking, activation, and effector activity [25,26].

However, a structural lung model without an active immune component is insufficient for modeling complex pathologies. The frontier in LOC evolution is the transition from structural biomimicry to functional immune competence. This involves not only co-culturing diverse cell types but engineering a dynamic environment that supports the viability, trafficking, and activation of immune cells (e.g., neutrophils, macrophages, T cells) within the device [27]. In a significant advance toward immunocompetence, Ringquist et al. advanced an LOC by incorporating vascular endothelial cells and immune cells, enabling the real-time visualization of cytokine storms during H1N1 infection [28,29]. Similarly, recent works have begun to address this by incorporating circulating immune cells into vascular channels to study asthma exacerbations [30]. These immunocompetent models have opened new avenues for disease modeling: Tan et al. constructed a tumor-on-a-chip model to simulate the tumor microenvironment (TME) and evaluate drug resistance [31], while Wang et al. created a lung–brain LOC to reveal how lung-derived inflammatory mediators drive COVID-19 neuropathology [32]. These advances underscore that immunocompetent models are essential for understanding how local tissue microenvironments dictate immune cell behavior and for evaluating the safety and efficacy of immunotherapies [33,34].

This review focuses on the engineering strategies and biological applications of immunocompetent LOC models (Figure 1). In this review, LOC is used as an umbrella term for microengineered lung models, encompassing both airway-on-chip and alveolus-on-chip systems. Where the biological requirements of proximal airway and distal alveolar compartments differ, these platforms are considered separately. Airway-on-chip models typically emphasize mucociliary epithelial function, mucus production and transport, airflow-associated cues, host responses to airway pathogens, neutrophilic inflammation, and disease processes relevant to asthma and chronic obstructive pulmonary disease (COPD). By contrast, alveolus-on-chip models focus on reconstruction of the thin alveolar–capillary barrier, interactions between type I and type II alveolar epithelial cells and capillary endothelium, cyclic mechanical stretch associated with breathing, alveolar macrophage function, and the gas-exchange interface. This distinction is particularly important when evaluating immunocompetence because the dominant epithelial, mechanical, and immune requirements differ substantially between proximal airway and distal alveolar microenvironments.

Airway-on-chip systems typically emphasize mucociliary differentiation, mucus production, airflow-associated exposure, and airway inflammatory responses, whereas alveolus-on-chip systems focus on the thin alveolar–capillary barrier, alveolar epithelial–endothelial interactions, gas-exchange-associated physiology, and breathing-related mechanical cues.

We define functional immunocompetence not simply by the presence of immune cells, but by the ability of a model to reproduce the immune functions required for its intended context of use. The relevant immune functions may range from epithelial innate signaling and leukocyte recruitment to antigen-specific adaptive responses, depending on the biological question being addressed. We first propose a functional grading framework for classifying immunocompetent LOCs. We then examine the engineering determinants of immune competence, including materials, mechanics, fluidics, extracellular matrix, and immune cell sourcing. Finally, we assess current applications in infection, fibrosis, and immunotherapy, distinguish chip-derived insights from those still dependent on conventional models, and discuss the major bottlenecks that limit progress toward adaptive immunity and translational use.

## 2. The Immunocompetence Grading Framework for Lung-on-a-Chip

The growing diversity of LOC platforms described as immunocompetent highlights the need for a shared evaluative language. Platforms that include immune cells can differ fundamentally in whether those cells are merely present or are actively executing immune functions. We therefore propose a four-tier framework in which classification is based on demonstrable immune function rather than cellular composition alone (Table 2). A platform that fails to meet the functional criteria of a given tier should be assigned to a lower tier regardless of the immune cell types included. The framework evaluates immune functions only insofar as they influence pulmonary biology, pathology, therapeutic response, or exposure outcomes (Figure 2).

Level 0 comprises immune-cell-free but immunoresponsive tissue models in which epithelial, endothelial, and stromal cells generate cytokine, chemokine, interferon, and stress-related inflammatory responses in the absence of dedicated immune cells. These systems are suited for barrier biology, epithelial toxicity, early inflammatory signaling, and selected inhalation toxicology studies, while capturing immunoregulatory signaling mediated by alveolar and endothelial cells.

Level 1 includes innate immune integration and requires evidence of active immune function, such as chemotaxis, phagocytosis, cytokine induction, or transendothelial migration. The survival of innate immune cells in co-culture is not sufficient; such models are better designated as Level 0^+^. The presence of dendritic cells alone also does not justify advancement beyond Level 1 unless antigen presentation and cognate T-cell priming are demonstrated.

Level 2 requires adaptive immune integration with antigen-specific function. Evidence may include T-cell activation, specific cytotoxicity, dendritic cell-mediated priming, antigen-specific class-switched antibody production, or clonal expansion. Because no single readout is fully sufficient, Level 2 classification should ideally be supported by multiple orthogonal assays. Platforms containing T or B cells but showing only non-specific activation should be classified as Level 1^+^, not Level 2.

Level 3 does not require all adaptive immune processes to occur within a single lung compartment. Rather, it encompasses functionally coupled multi-compartment or multi-organ systems that reproduce lung-immune interactions relevant to adaptive immunity.

The framework should not be interpreted as a universal hierarchy of model quality; rather, it indicates increasing breadth of immune functionality.

This framework is intended to discriminate between nominal and functional immunocompetence. For example, a platform containing THP-1-derived macrophages and PBMC-derived T cells stimulated with anti-CD3/CD28 beads includes both innate and adaptive cell types, yet without antigen-specific priming it does not exceed Level 1^+^. Likewise, total IgG secretion without antigen specificity should not be interpreted as adaptive immune competence. Throughout this review, we apply this framework to clarify what current platforms truly achieve, what functions remain absent, and which disease questions can be addressed at each level.

## 3. Construction Strategies of Immunocompetent Lung-on-a-Chip

Developing a LOC platform capable of modeling immune responses requires more than simply co-culturing cells. It demands the precise engineering of a microenvironment that supports the recruitment, infiltration, and activation of immune cells. In this section, we focus on the engineering determinants that define the attainable level of immunocompetence, including material selection, structural design, and dynamic mechanical cues.

### 3.1. Foundations and Materials for Lung-on-a-Chip

LOCs are constructed at the intersection of microfluidics, biomaterial science, and cell biology [35,36], utilizing miniaturized chip platforms to simulate the complex structure and physiological functions of lung tissue [21,37,38], as shown in Figure 3a. The core lies in employing advanced manufacturing processes and design principles to construct environments containing multiple cell types and their interactions, achieving a highly simulated ecological environment for complex lung tissues [39,40,41]. These structural advances made it possible to model barrier physiology and lung injury in vitro, but they also revealed that material choice is not a neutral design variable. In immune-focused systems, it directly shapes the fidelity of signaling, cell behavior, and functional readouts.

PDMS remains the most widely used material because of its optical transparency, biocompatibility, and elasticity. However, it presents several limitations for immunological studies. Its hydrophobic surface can adsorb small molecules, including drugs and cytokines, thereby distorting measured inflammatory responses [42]. Surface modifications such as parylene coating can reduce this effect [43]. Beyond molecular adsorption, an underappreciated challenge specific to immunological studies is the leaching of uncrosslinked PDMS oligomers [44], which can directly interact with cell membranes and alter cellular behavior. Immune cells are particularly sensitive to such biophysical perturbations, as their activation state can be modulated by minute changes in their physicochemical environment [45]. This concern is especially acute for long-term immune co-culture experiments, where cumulative oligomer exposure may confound functional readouts. A mechanical challenge also persists: standard PDMS exhibits an elastic modulus in the megapascal (MPa) range, which differs significantly from the kilopascal (kPa) range characteristic of native lung tissue. This mechanical mismatch is relevant because matrix stiffness can modulate immune cell behavior and functional responses [46].

Beyond PDMS and hydrogel-based platforms, thermoplastics are increasingly used in LOCs and broader MPS technologies. Materials such as cyclic olefin polymers/copolymers (COP/COC), polycarbonate, polymethyl methacrylate (PMMA), and polystyrene can provide advantages for scalable manufacturing, device standardization, and reduced absorption of some hydrophobic compounds relative to PDMS. Their compatibility with injection molding and other high-throughput fabrication approaches also makes them attractive for translation and commercialization. However, thermoplastic platforms may require additional surface modification, bonding strategies, or engineered gas exchange. Their greater rigidity may also constrain applications in which substantial membrane deformation is specifically required, although rigidity itself is not necessarily a limitation for LOC systems designed for other biological endpoints.

This distinction is particularly important when considering the membrane configuration of pulmonary LOC systems. Porous membranes used in LOC systems can be broadly divided into mechanically deformable and relatively rigid configurations. Flexible membranes, including elastomeric membranes, enable cyclic deformation and are particularly valuable for modeling breathing-associated mechanical strain and mechanobiological responses. However, membrane flexibility is not a universal requirement for pulmonary LOC systems. Many platforms employ rigid porous membranes when the primary objectives are epithelial–endothelial coculture, barrier transport, vascular perfusion, immune-cell recruitment, or drug-exposure studies. Accordingly, membrane material and mechanical configuration should be selected according to the intended biological question, rather than membrane deformability being considered an intrinsic requirement of LOC design.

These limitations have driven increasing interest in alternative materials. Hydrogels such as GelMA, collagen, and fibrin provide a hydrated and permeable matrix that more closely resembles the native extracellular environment and better supports the exchange of signaling molecules [47,48]. To more closely reproduce the architecture of the native pulmonary microenvironment, LOC platforms increasingly employ micro- and nanoscale fabrication strategies. By controlling the thickness of PLGA nanofiber membranes to approximately 3 µm, the physiological thickness of the alveolar respiratory membrane can be replicated, offering a more realistic barrier than thicker PDMS membranes [49,50]. Similarly, Maibohm et al. utilized the polymer SZ2080 combined with two-photon polymerization to construct a 3D structured LOC spanning subcellular to tissue scales. The low non-specific adsorption and low autofluorescence of this material facilitate real-time observation of cell behavior while minimizing interference with biological readouts [51]. Importantly, material properties are not immunologically neutral. Material selection may influence protein adsorption, cytokine availability, cell adhesion, and inflammatory signaling, and should therefore be considered not only as an engineering choice but also as a potential regulator of immune behavior.

For immunocompetent lung chips, material selection should therefore be evaluated not only by fabrication convenience or general biocompatibility, but also by its impact on immune interpretability. Key considerations include cytokine adsorption, oligomer leaching under prolonged perfusion, mechanical matching to native lung tissue, and optical suitability for real-time immune tracking. At present, hydrogel-based or hybrid designs that combine PDMS microfluidics with hydrogel cell compartments appear to offer a practical compromise. However, the immunomodulatory effects of most alternative materials remain insufficiently characterized, and systematic benchmarking across standardized immune assays is still needed.

### 3.2. Structural Modules of LOC and Their Immune

An LOC replicates the complex microenvironment of lung tissue by integrating specific structural modules that mimic the organ’s architecture and physiological dynamics. Crucially, each of these modules is not merely a structural feature, but rather a functional regulator of immune behavior. The engineering decisions underlying the design of the airway chamber, vascular channel, and porous membrane directly determine whether immune cells can perform their surveillance and effector functions. Below, we examine how each element contributes to specific immune processes and highlight instances in which these designs have led to biological insights unattainable in conventional models.

#### 3.2.1. Alveolar/Airway Chamber

The airway compartment reproduces the luminal side of the respiratory interface by exposing epithelial cells to air under an air–liquid interface (ALI), thereby preserving epithelial polarity, barrier integrity, and mucociliary function [52]. As shown in Figure 3b, Curved or sac-like geometries can further approximate alveolar architecture and support prolonged epithelial differentiation. However, the time required to establish a mature epithelial phenotype introduces an important constraint for subsequent immune-cell integration: epithelial differentiation and barrier maturation may require days to weeks, whereas some primary immune-cell populations require fresh isolation, have limited ex vivo longevity, or may undergo functional changes following cryopreservation or prolonged culture. These features are particularly important for modeling the earliest stages of host–pathogen interaction, where epithelial sensing, cytokine release, and barrier disruption initiate downstream immune responses. Huh et al. integrated epithelial, endothelial, and immune components to reproduce organ-level barrier responses to bacterial toxins [29]. Building on this, Zhang et al. incorporated an extracellular matrix (ECM) into the airway compartment, creating a 3D inflammation model. By establishing controllable chemical gradients across the ALI, they achieved high-resolution imaging of neutrophil migration, thereby reaching immunocompetence Level 1 [53]. These models provide a robust platform for assessing host immune responses and barrier dysfunction during infection.

Beyond barrier integrity, reconstitution of mucus production is a key functional benchmark for pulmonary mucosal immunity. ALI-cultured epithelia can differentiate into goblet cells, which trap pathogens and support mucociliary clearance. Dysregulated mucus secretion, a hallmark of asthma, COPD, and cystic fibrosis, has been modeled in LOC platforms [54], where IL-13 drives goblet cell metaplasia and hypersecretion, and mechanical stretch modulates this response, linking biophysical cues to mucosal output [55]. Future platforms targeting Level 2 or 3 immunocompetence should incorporate quantitative mucus readouts (e.g., mucin concentration, viscoelasticity, ciliary beat frequency) as functional endpoints in allergic or chronic infection models.

#### 3.2.2. Vessel-like Cultivation Chamber

Complementing the airway chamber, the vascular channel introduces endothelial cells and fluid flow to mimic the pulmonary capillary network. This compartment provides continuous hemodynamic stimuli, including fluid shear stress (FSS), which is vital for endothelial homeostasis and immune cell recruitment. Yang et al. optimized channel geometries to reproduce physiological FSS variations, demonstrating how blood flow dynamics regulate endothelial phenotypes [56]. In a separate study, they further customized branched microchannel networks to mimic the capillary architecture surrounding alveolar sacs, ensuring cells were subjected to physiologically relevant mechanical loads (Figure 3c) [57]. To facilitate immune interactions, van Os et al. designed a parallel channel layout separated by a fibrin hydrogel barrier. This configuration allowed for the precise control of fluid hemodynamics and chemokine gradients, enabling real-time tracking of immune cell migration across the vascular endothelium during pulmonary inflammation [58].

A further challenge, particularly for donor-matched immunocompetent LOCs, is the temporal synchronization of tissue maturation and immune-cell availability. Epithelial and endothelial compartments may require days to weeks of differentiation and conditioning before stable barrier and vascular phenotypes are established, whereas primary immune cells may need to be isolated close to the time of use, may exhibit limited ex vivo persistence, or may undergo phenotypic and functional alterations after freeze–thaw cycles or prolonged culture. This creates a practical problem for autologous models: functionally intact, donor-matched immune cells must be introduced only after the corresponding tissue compartments have reached the required state of maturity. Potential strategies include cryobanking PBMCs collected from the same donor, staged immune-cell introduction after tissue maturation, repeated replenishment of short-lived immune populations, parallel donor-specific banking of structural and immune-cell sources, the use of iPSC-derived immune cells, and modular injection through the vascular channel at defined experimental time points. These approaches are not functionally equivalent. Cryopreservation can alter selected immune phenotypes or responses, whereas iPSC-derived immune cells offer scalability and genetic matching but may not fully reproduce the phenotypic and functional diversity of adult primary immune cells. Therefore, temporal synchronization should be treated as an explicit design variable in immunocompetent LOCs and validated using predefined measures of post-thaw viability, phenotype, activation state, trafficking behavior, and effector function rather than inferred solely from the presence of immune cells.

#### 3.2.3. Flexible Porous Membrane

Connecting the epithelial and endothelial compartments is a flexible porous membrane, which serves as a structural scaffold for bilayer cell culture and a mechanical transducer. Crucially, this membrane mimics the basement membrane’s permeability, physically separating the air and blood phases while permitting the diffusion of cytokines and the exchange of metabolic substances [59,60], as well as the transmigration of immune cells [61]. Utilizing the tunable elasticity of PDMS, the membrane can undergo periodic deformation driven by pneumatic actuation, simulating the cyclic expansion and recoil of alveoli during breathing [62,63]. In mechanically active LOC systems, the magnitude and distribution of strain experienced by lung cells are governed by factors including membrane stiffness and thickness, applied pressure or vacuum, device geometry, and actuation parameters [64,65]. Guenat et al., for example, used an ultrathin porous membrane to apply defined tensile strain and showed how mechanical loading contributes to lung injury and fibrotic remodeling [66]. Qiu et al. further advanced this approach by developing a double-layer device (Figure 3d) in which membrane deformation amplitude and frequency could be precisely tuned to reproduce the mechanical dynamics of respiration, influencing both cell metabolism and inhalant deposition [65].

#### 3.2.4. Fluidic Design and Functional Integration

The functionality of an LOC relies on sophisticated fluidic design to manipulate the microenvironment with high spatiotemporal resolution. Precise fluid control allows researchers to simulate both stable physiological blood flow and the altered hemodynamics associated with inflammation or infection [67]. Wang et al. utilized live imaging within a microfluidic device to reveal that the narrow constriction of pulmonary capillaries forces neutrophils to deform, identifying the mechanosensitive channel PIEZO1 as a key regulator of pulmonary vascular homeostasis and immune cell migration [68]. This finding represents one of the few examples in which a LOC platform has directly elucidated a mechano-immunological mechanism, specifically, how mechanical forces sensed through PIEZO1 reprogram neutrophil phenotype and migration behavior. Beyond mechanics, fluidic systems enable the transport of aerosols. Artzy-Schnirman et al. integrated a 3D-printed airway tree with an ALI chip to reproduce the complex trajectories of inhaled particles, bridging the gap between macro-scale respiration and micro-scale cellular responses (Figure 3e) [69]. Additionally, surface modification technologies within these channels can further regulate immune cell adhesion and behavior, which is crucial for simulating the pulmonary immune microenvironment [65]. Finally, the integration of micro-pumps and valves facilitates the automated delivery of immunomodulators, while embedded sensors allow for real-time monitoring of biomarkers [46,70,71]. By precisely regulating flow parameters, uniform and continuous fluid flow can simulate stable physiological states, while altering flow velocity and pressure can recreate immune responses under pathological conditions like infection or inflammation [72]. These LOC implementations successfully replicate complex alveolar/airway chamber physiological models, enabling researchers to conduct real-time observation of complex immune behaviors, including neutrophilic inflammatory responses and transendothelial migration [73].

The structural elements of LOCs are not passive scaffolds but active determinants of immune competence. The design choices made at the level of chamber geometry, membrane properties, and fluidic control directly constrain the level of immunocompetence a platform can achieve: simple bilayer configurations support Level 0–1, while advanced designs incorporating dynamic mechanical cues, chemokine gradient control, and mechanosensitive readouts are prerequisites for progressing toward Level 2–3. For this reason, engineering decisions should be guided from the outset by the target immunocompetence level and its corresponding functional readouts.

**Figure 3:**
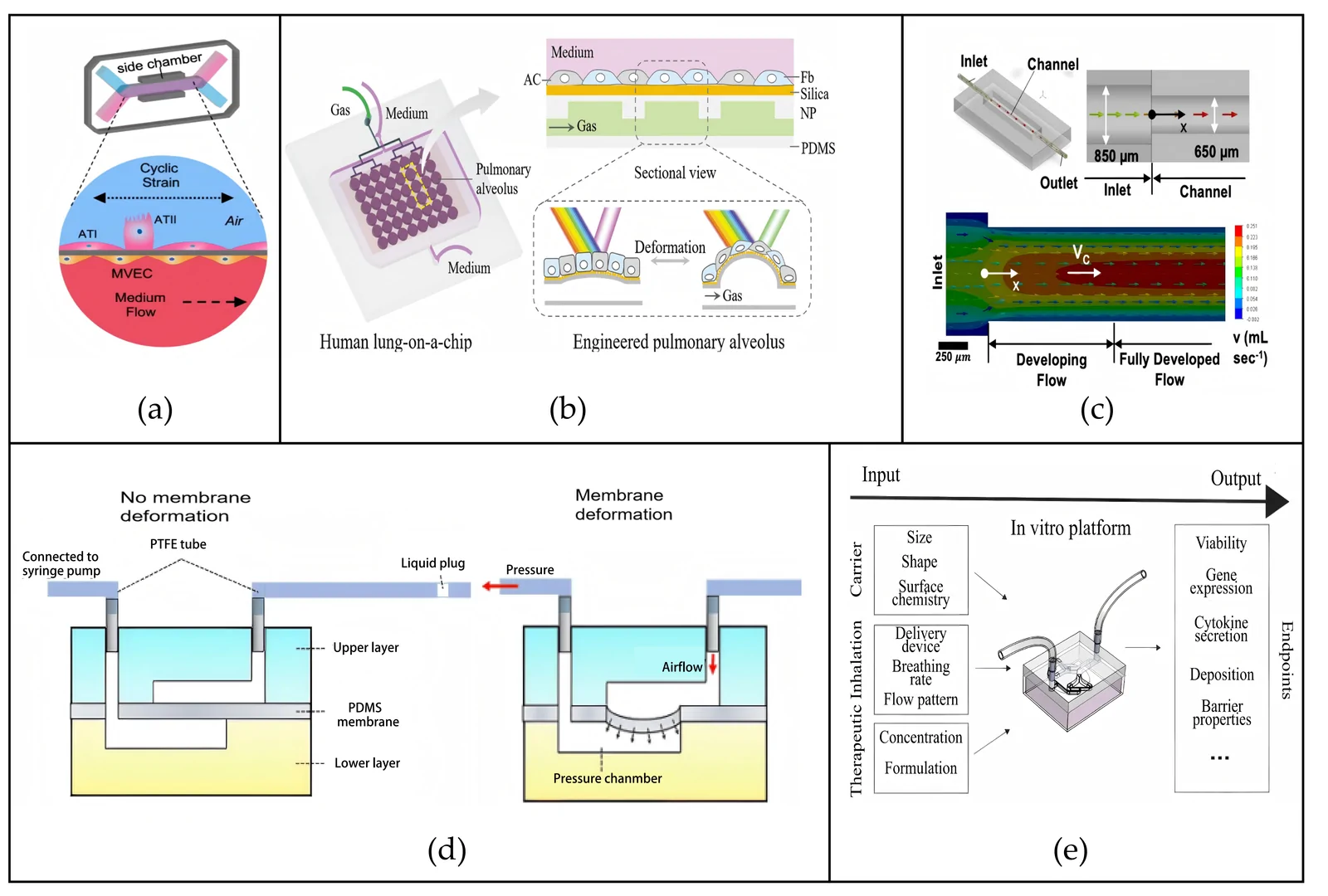
Structural and functional elements of an LOC. (**a**) Canonical air-liquid barrier architecture, in which lung epithelial cells line the alveolar lumen and endothelial cells line the vascular channel. Ref. [21]. (**b**) Breathing chip design that reproduces cyclic mechanical stimulation associated with respiration. Ref. [52]. (**c**) Vascular chip configuration modeling hemodynamic forces in the pulmonary microcirculation. Ref. [57]. (**d**) Stretchable alveolar chip incorporating a central elastic PDMS membrane and a pressure-actuated chamber to simulate breathing-associated tissue deformation. Ref. [65]. (**e**) Integrated in vitro inhalation platform coupling carrier design, inhalation parameters and formulation variables with biological readouts, including cell response, barrier function and aerosol deposition. Ref. [69].

### 3.3. Biological Microenvironment and Cellular Components of LOC

Each engineering determinant sets a ceiling on attainable immunocompetence. Cell lines and simple bilayer PDMS devices restrict platforms to Level 0–1. PBMCs and standard perfusion can support Level 2 only when antigen-specific T-cell responses are functionally validated. Primary tissue-resident immune cells, immunomodulatory ECM, and spatially organized niches are prerequisites for Level 3. Beyond these physical constraints, biological design dictates how faithfully a model recapitulates native pulmonary immunity. In immunocompetent lung chips, the achievable immune function depends not only on which cell types are included, but also on how faithfully their abundance, source, spatial organization, matrix context, and temporal dynamics reproduce the in vivo immune niche.

#### 3.3.1. Cellular Composition and Stoichiometry

The physiological relevance of a LOC model is fundamentally determined by the fidelity of its cellular composition and the stoichiometric ratios of interacting cell types [74]. The lung represents a complex ecosystem where alveolar epithelial cells, endothelial cells, and immune cells (macrophages, T cells, and dendritic cells) coexist in specific proportions to maintain homeostasis [75]. Within the alveolar epithelial compartment, model design must additionally consider the distinct functions of alveolar type I (AT1) and type II (AT2) epithelial cells. AT1 cells cover the majority of the alveolar surface area and form the thin epithelial component of the gas-exchange barrier, whereas AT2 cells produce pulmonary surfactant and contribute to epithelial maintenance and regeneration following injury. Consequently, the relative representation, differentiation state, and phenotypic stability of AT1- and AT2-like populations can substantially influence the physiological fidelity and functional interpretation of alveolar LOC systems. These differences are also relevant to immune modeling, as epithelial subtype and differentiation state can influence cytokine secretion, injury responses, and epithelial-immune crosstalk. Beyond cell-type identity, recapitulating physiologically relevant cellular stoichiometry is also important for modeling authentic immune responses. Achieving cell-type-appropriate stoichiometry in an LOC remains a significant engineering challenge, particularly when incorporating multiple immune lineages simultaneously [76]. Deviations from these physiological ratios can inadvertently skew immune signaling, leading to artifacts in experimental data. Therefore, recapitulating the native cellular stoichiometry is crucial for modeling authentic immune responses (Figure 4a) [77]. For instance, establishing a functional air–liquid interface requires not only epithelial barrier formation but also the integration of resident immune cells at physiological densities to mimic host defense mechanisms against pathogens or allergens [78,79,80]. The importance of this balance was demonstrated by Kim et al., who modulated the ratio of macrophages to T cells within a lung cancer organoid. Although not a lung-on-chip system, the study demonstrated that a specific cellular stoichiometry is required to trigger T cell-mediated inflammatory responses effectively [81]. Similarly, Gultom et al. highlighted the quantitative role of endothelial cells in vascular inflammation. By precisely tuning the endothelial cell fraction, the researcher modeled the progression of vascular inflammation, underscoring that cell ratio represents a critical variable in defining the pathological state model [82]. Cellular composition and stoichiometry are therefore not merely background design variables but active determinants of immune output and key dimensions of immunocompetence across the Level 0–3 framework.

Within the proposed immunocompetence framework, cellular composition and stoichiometry can also be interpreted across Levels 0–3. Level 0 models contain no dedicated immune cells and are composed primarily of epithelial, endothelial, and/or stromal compartments. Level 1 systems introduce one or a limited number of innate immune populations, such as resident macrophages or transiently perfused neutrophils, but their abundance is often determined by experimental feasibility rather than by physiologically justified ratios. Level 2 models incorporate multiple immune populations relevant to the disease or biological question, with explicit rationale for immune-to-structural cell ratios and validation of immune-cell dynamics or function. Level 3 extends beyond static numerical matching by reproducing physiologically justified and dynamically regulated cellular composition, including recruitment, expansion, differentiation, and the coexistence of resident and circulating immune niches. Importantly, pulmonary cellular stoichiometry is not fixed in vivo but varies with anatomical region, age, disease state, and stage of infection. Therefore, Level 3 should emphasize context-dependent and dynamically regulated cellular composition rather than exact replication of a single numerical cell ratio.

#### 3.3.2. Epithelial Cell Sources

The choice of epithelial cell source is a parallel determinant of LOC fidelity, particularly in alveolar models where cellular maturity, phenotypic stability, and donor specificity can directly affect tissue function and epithelial–immune interactions.

A major consideration in alveolar LOC design is the choice between primary human alveolar epithelial cells and induced pluripotent stem cell (iPSC)-derived alveolar populations. Primary cells provide direct access to adult and potentially donor-specific pulmonary phenotypes, but their availability is limited, and maintenance of stable alveolar phenotypes during expansion and prolonged culture remains challenging. In particular, primary alveolar epithelial cells may undergo phenotypic changes under in vitro culture conditions, complicating long-term model establishment.

iPSC-derived alveolar cells provide an alternative route to scalable and potentially patient-specific cell production and offer opportunities to generate multiple cell types from a common genetic background. However, their use introduces other challenges, including differentiation efficiency, batch-to-batch variability, cellular heterogeneity, and the extent to which differentiated cells reproduce mature adult alveolar phenotypes.

Primary and iPSC-derived alveolar epithelial cells should therefore be regarded as complementary rather than universally interchangeable cell sources, with the optimal choice depending on the required degree of cellular maturity, donor specificity, experimental duration, scalability, and intended context of use.

#### 3.3.3. Immune Cell Sources

In parallel with epithelial cell sourcing, the source of immune cells places a practical and biological ceiling on the level of immunocompetence that a platform can credibly claim. Four sources are currently most common, each with distinct advantages and limitations.

THP-1 monocytic leukemia cells are the most widely used source owing to their unlimited proliferative capacity and operational convenience [83]. However, THP-1 cells carry well-documented functional deficits relevant to pulmonary immune modeling: their NLRP3 inflammasome activation pathway differs fundamentally from that of primary monocytes [84], and their chemotactic behavior shows reduced directionality with altered receptor expression compared to primary cells. THP-1 cells are therefore best suited for high-throughput initial screening rather than mechanistic conclusions. Consequently, THP-1-based platforms are inherently restricted to Level 1 immunocompetence.

Peripheral blood mononuclear cells (PBMCs) offer the advantage of human origin with relative accessibility and retain functional competence in cytokine secretion and cytotoxicity assays. However, PBMCs are fundamentally limited in modeling tissue-resident immunity; circulating monocytes display a distinct surface profile (CD14+) compared to alveolar macrophages, which are imprinted by the local TGF-β-rich and GM-CSF-rich pulmonary microenvironment to express CD169, PPARγ, and high levels of CD206 [85]. PBMC-based platforms can, in principle, achieve Level 2 immunocompetence; antigen-specific T cell responses and cytotoxicity are preserved, but the systemic rather than tissue-resident character of the immune response must be explicitly acknowledged as a model limitation.

Primary tissue-resident immune cells, such as alveolar macrophages, lung-resident dendritic cells, and bronchial lavage-derived lymphocytes, provide the highest biological fidelity and are indispensable for achieving higher immunocompetence levels. However, their use is constrained by source scarcity and substantial donor- and disease-dependent variability [86], which is both a source of experimental noise and a biologically meaningful dimension of personalized modeling. Platforms employing primary tissue-resident immune cells are the only current systems capable of supporting Level 2–3 immunocompetence with biological credibility, as they alone preserve the epigenetic memory of prior exposures, the tissue-specific phenotypic imprinting, and the spatial organization required for authentic adaptive and lymphoid-level immune function.

iPSC-derived immune cells (macrophages, NK cells, and T cells) offer the prospect of genetic manipulation, isogenic autologous pairing with iPSC-derived epithelial and endothelial cells, and scalability. However, their functional maturity remains an active area of debate: transcriptomic comparisons indicate that iPSC-derived macrophages more closely resemble yolk-sac-derived primitive macrophages than adult alveolar macrophages [87]. iPSC-derived immune cells currently fall into an intermediate category. They hold the theoretical potential to support Level 2 or 3 immunocompetence in fully autologous platforms, but their functional equivalence to primary tissue benchmarks has not yet been demonstrated.

Beyond macrophages, each immune lineage imposes a distinct ceiling on attainable immunocompetence. PBMC-derived T cells can, in principle, support Level 2 functions such as antigen-responsive cytotoxicity and cytokine production, but current lung-chip evidence still captures these outputs more readily than tissue-resident memory or durable recall behavior [88]. Neutrophils are especially informative for Level 1 studies of chemotaxis, adhesion, and transendothelial migration, yet their short lifespan and sensitivity to continuous perfusion limit their utility in prolonged multi-step immune circuits [89]. NK cells are well suited to modeling early cytotoxic surveillance, but in most current chip systems their function remains closer to Level 1 or Level 1^+^, because the stromal and cytokine cues required for licensing and sustained activation are not yet robustly reproduced [90]. Dendritic cells remain the decisive lineage for Level 2 advancement: monocyte-derived dendritic cells are useful for exploratory antigen-presentation studies, but Level 2 classification should be reserved for platforms that demonstrate antigen-specific T-cell priming through multiple orthogonal functional readouts [91]. In this context, primary lung dendritic cells remain the strongest biological benchmark, even though their routine integration into scalable chip workflows is still not feasible.

Taken together, immune cell sourcing should be treated as a formal classification variable rather than a technical detail. Cell lines are compatible with Level 1 discovery; blood-derived primary cells enable stronger Level 1 and selective Level 2 applications; tissue-resident primary cells provide the most credible path to Level 2–3; and iPSC-derived systems remain promising only when maturity is explicitly benchmarked. A field-wide benchmarking effort across common chip architectures, using shared functional assays tied to the Level 0–3 framework, would substantially improve comparability and reduce overstatement of immunocompetence.

#### 3.3.4. Spatial Organization and Intercellular Communication

Beyond cellular composition, the spatial organization defined by the 3D arrangement, compartmentalization, and physical adjacency of cells dictates both the mode and efficiency of intercellular communication [92]. The relative positioning, compartmentalization, and physical adjacency of epithelial, endothelial, stromal, and immune cells govern whether signaling occurs through direct contact, directed migration, or soluble mediators alone. This is particularly important because some immune processes, such as antigen presentation and immune synapse formation, require sustained cell–cell contact, whereas others depend primarily on polarized cytokine gradients.

Accordingly, an LOC can be used not only to reproduce multicellular organization but also to dissect contact-dependent from contact-independent signaling. Direct physical contact is often a prerequisite for specific immune activation events. Marzban et al. utilized DNA nanotechnology to program the spatial assembly of immune cells, demonstrating that specific geometric configurations between T cells and antigen-presenting cells significantly enhanced immune synapse formation and signal transmission [93]. Conversely, to isolate contact-independent mechanisms, Yadav employed a transwell-based ALI system where macrophages and epithelial cells were physically separated by a porous membrane. This topology revealed that macrophages could trigger epithelial proliferation via soluble factors upon carbon nanotube sensing, independent of physical contact [94]. Tran et al. compared contact versus non-contact co-cultures of tumor cells and fibroblasts, finding that direct physical interaction was positively correlated with tumor proliferation, a phenomenon absent in purely paracrine models [95]. The ability to experimentally dissect contact-dependent from paracrine signaling is a prerequisite for advancing from descriptive Level 1 co-cultures to mechanistic Level 2 platforms.

Furthermore, maintaining specific spatial compartmentalization is vital for directional signaling during infection. Gu et al. utilized a humanized LOC to spatially segregate epithelial cells, B cells, and T cells, revealing that epithelial infection with Influenza A virus triggers a type I interferon response that activates distal immune cells [96]. Similarly, Plebani et al. co-cultured bronchial epithelial and microvascular endothelial cells in separate compartments to model cystic fibrosis. This spatial arrangement allowed researchers to observe the directional recruitment of neutrophils driven by IL-8 gradients across the endothelial barrier in response to *P. aeruginosa* infection (Figure 4b), a process that relies heavily on the distinct polarization of the epithelial–endothelial interface [78]. Such long-term, dynamic simulation of immune responses is closer to in vivo experimental outcomes, suggesting that this approach holds promise for surpassing existing in vitro immunology studies and offers significant value for understanding the immune mechanisms of long-term, chronic lung diseases like asthma and lung cancer [97]. Such architectures remain mostly within Level 1, but they establish the organizational logic required for more advanced adaptive interactions.

#### 3.3.5. ECM as an Instructive Immune Niche

The extracellular matrix (ECM) is not merely a scaffold but an instructive niche that regulates immune cell migration, differentiation, retention, and signaling through both biochemical composition and mechanical properties [98]. ECM composition varies substantially across pulmonary compartments and creates distinct microenvironments that differentially influence immune cell behavior. Kitamura et al. demonstrated that lung-derived decellularized ECM sheets could direct embryonically derived stem cell differentiation toward lung-like phenotypes, illustrating the instructive capacity of tissue-specific matrix (Figure 4c) [99]. Innovative engineering approaches have also enabled the dynamic manipulation of the ECM. Humayun et al. separated airway epithelial and smooth muscle cells with a removable hydrogel layer. This design facilitated paracrine signaling while maintaining spatial isolation, and the subsequent removal of the hydrogel allowed for the independent analysis of each cell type. While this model successfully mimicked epithelial–mesenchymal crosstalk, it also highlighted the challenges in replicating complex 3D architectures, such as the ring-like smooth muscle configuration seen in vivo [79]. We argue that the next generation of immunocompetent LOC platforms must treat ECM composition as an independent experimental variable rather than a passive scaffold. Achieving Level 3 immunocompetence, in which organized lymphoid structures form and are maintained, will likely require spatially patterned ECM with compartment-specific biochemical cues, a capability that remains a significant materials engineering challenge. Beyond biochemical composition, the choice of ECM material itself carries practical implications for immune readouts: natural ECM materials may exhibit batch-to-batch variability and contain immunogenic components that can trigger unintended inflammatory signaling, complicating the interpretation of immune functional readouts. Decellularized human-derived or synthetic matrices may offer improved consistency, but their immunomodulatory properties require systematic characterization.

#### 3.3.6. Temporal Dynamics and Culture Longevity

Immune responses are inherently dynamic processes that evolve over time, ranging from rapid acute inflammation to chronic remodeling. Consequently, an LOC must support long-term culture and monitoring to capture these temporal shifts [100]. Goksel et al. achieved a 72 h dynamic simulation of PM2.5 exposure in an airway chip, revealing time-dependent recovery kinetics that would be invisible in endpoint assays [101] (Figure 4d). Beyond duration, the periodicity of immune events is critical. Noursadeghi et al., utilizing a SARS-CoV-2 human challenge model, deciphered the temporal sequencing of immune gene expression, identifying a precise transcriptional switch that discriminates early from late phases of respiratory viral infection [102]. Translating such temporal resolution to LOC platforms would enable the identification of therapeutic windows for immunomodulatory intervention. McGinnis et al., using a mouse model of breast cancer lung metastasis combined with single-cell analysis, discovered a time-dependent IGF1–IGF1R communication axis between alveolar macrophages and neutrophils, revealing the periodic temporal dynamics of pulmonary immune cell interactions [103]. Most LOCs do not yet reproduce these processes directly, but they provide an important foundation for doing so. Capturing this temporal dimension will be essential not only for determining whether an immune response occurs, but also for resolving when it begins, how it propagates, and whether it resolves or progresses toward pathology.

**Figure 4:**
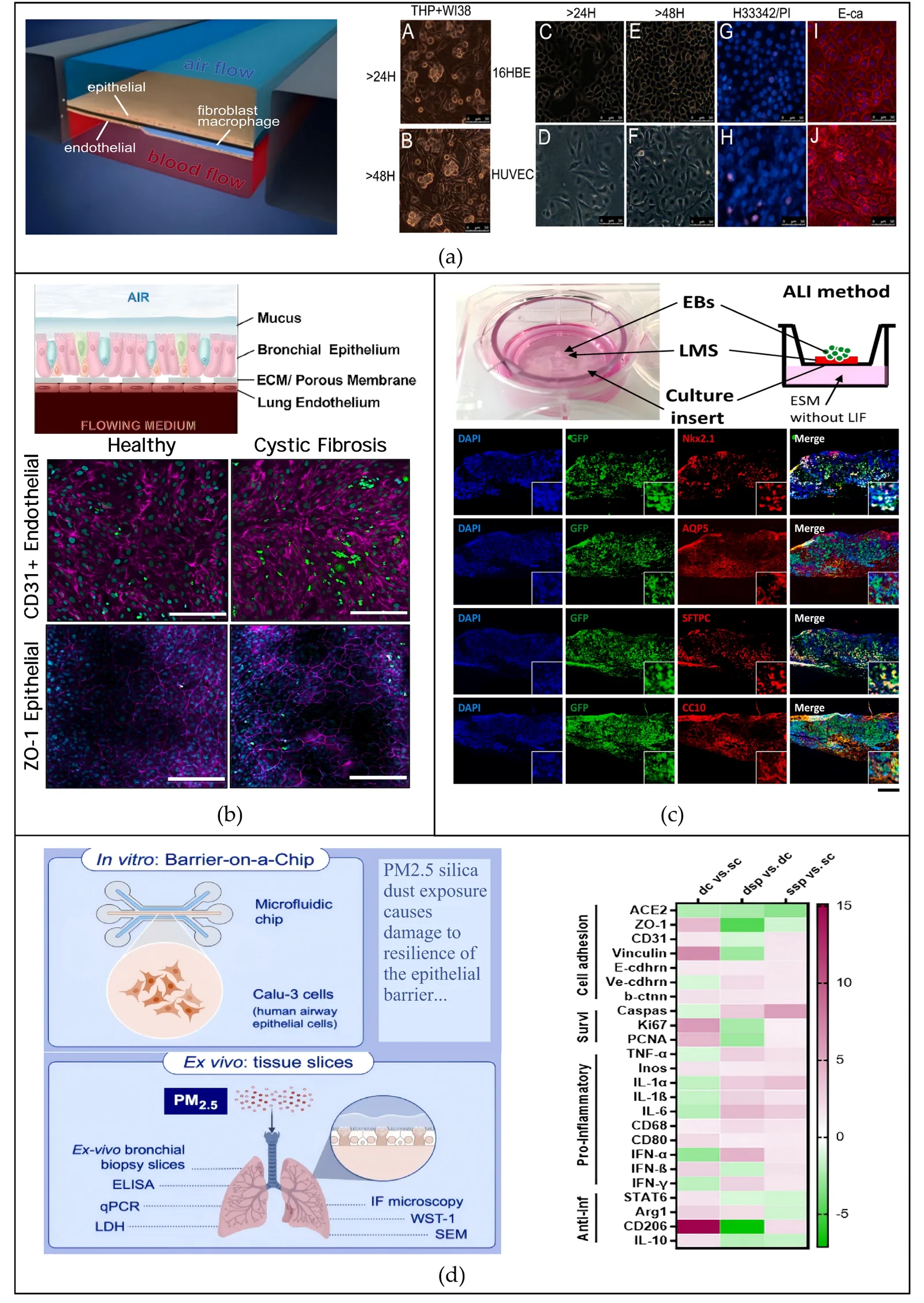
Multidimensional determinants of immune microenvironment construction in lung-on-a-chip platforms. (**a**) (A,B) Morphological features of fibroblast WI38 and macrophage THP-1 cells cultured in the microfluidic chip after 24 h (top panel) and after 48 h (bottom panel). (C–F) Morphological features of bronchial epithelial 16HBE cells (top panel) and microvascular endothelial HUVEC cells (bottom panel) cultured in the microfluidic chip. (G,H) Cell viability assessed by Hoechst/PI staining. (I,J) E-cadherin expression (red) and nuclear DAPI stain (blue). The lung-on-a-chip platform adopts spatially organized multicellular co-culture: bronchial epithelial cells (16HBE) occupy the air channel, while endothelial cells (HUVEC), fibroblasts (WI38) and macrophages (THP-1) are arranged in the perfused compartment to establish a structured immune microenvironment. Ref. [77]. (**b**) Distinct endothelial–epithelial signaling under healthy and cystic fibrosis conditions, with neutrophil adhesion, epithelial barrier disruption, and inflammation-driven trans-tissue migration in the disease state. (Scale bar, 200 μm) Ref. [78]. (**c**) Lung matrix-supported differentiation of mouse embryonic stem cells, with expression of lung-associated markers including Nkx2.1, AQP5, SFTPC, and CC10. (Scale bar: original images, 400 μm; inset images, 100 μm) Ref. [99]. (**d**) Divergent response trajectories in PM2.5 exposure under dynamic versus static culture conditions, highlighting the importance of temporal modeling. Ref. [101].

Achieving immunocompetence above Level 1 requires the simultaneous optimization of cellular fidelity, immune cell source selection, spatial organization, ECM composition, and temporal dynamics. These dimensions are interdependent. For example, the choice of immune cell source (PBMCs vs. tissue-resident) directly constrains the spatial organization that can be modeled, and ECM composition determines the temporal stability of immune cell phenotypes. Progress beyond Level 1 will therefore require coordinated optimization across these dimensions rather than incremental addition of immune cell types alone.

### 3.4. The Adaptive Immune Bottleneck

Before moving to disease-specific applications, one unresolved issue must be stated explicitly: adaptive immune integration remains the major bottleneck in lung-on-a-chip engineering [30]. Although Level 2 is widely regarded as the next decisive milestone, it has been achieved only rarely and usually in partial form [28]. The difficulty lies not simply in adding lymphocytes, but in reconstructing the sequential logic of adaptive immunity within devices that were originally optimized for epithelial–endothelial homeostasis.

The first constraint is antigen-presentation quality control. Productive T-cell priming requires professional antigen-presenting cells, typically dendritic cells, in a maturation state that balances co-stimulatory signaling with appropriate cytokine output. In practice, however, the ex vivo differentiation protocols and media conditions required for dendritic-cell maturation and lymphocyte support are often poorly compatible with those needed to maintain lung epithelial and endothelial tissues [104]. A second constraint is the conflict between perfusion and synapse stability. Adaptive activation depends on prolonged, stable APC–T-cell contact, whereas the continuous flow required to preserve nutrient delivery, shear stress, and barrier physiology can destabilize precisely these interactions [30]. A third constraint is the architectural threshold for B-cell immunity. Class-switch recombination, affinity maturation, and durable antibody responses require organized follicular microenvironments, Tfh support, and follicular dendritic cell-like networks, none of which has yet been convincingly reconstituted in pulmonary chip systems. A fourth constraint is donor matching. Allogeneic co-cultures readily trigger mixed lymphocyte reactions that obscure antigen-specific responses [105], whereas fully autologous platforms require coordinated access to epithelial, endothelial, and multiple immune cell populations from the same donor, which is often impractical in real clinical settings [106,107]. Whether all cellular components must be donor-matched, however, depends on the intended immune function and context of use. A fifth constraint is temporal stability. Adaptive responses typically unfold over weeks rather than days, yet most complex lung-chip co-cultures remain stable only over comparatively short time windows, limiting the observation of clonal expansion, durable effector programs and memory-like states [108].

These barriers are not independent. Reducing flow may stabilize cell–cell contact but compromise tissue viability; extending culture duration increases the demands on matrix stability, media compatibility and donor-matched cell supply; adding architectural complexity for B-cell support further reduces throughput and reproducibility. For this reason, progress to Level 2 should not be viewed as a linear extension of Level 1 by incremental cell addition. It is a coupled systems-engineering problem that will likely require protected immune niches, modular media strategies, longer-lived matrices, and more robust autologous or human leukocyte antigen (HLA)-matched design pipelines [41].

### 3.5. Throughput–Biomimicry Trade-Offs and a Design Decision Framework

The demand for high-throughput screening competes directly with the drive toward increasing physiological fidelity. High-content screening requires standardization, simplification, and parallelization, attributes that favor minimal cell types, defined media, and short culture durations. High biomimicry, by contrast, demands complex cellular composition, patient-specific parameters, dynamic mechanical stimulation, and extended culture periods to capture chronic immune processes. This trade-off is not a binary choice but a continuous spectrum that must be navigated according to the experimental objective.

Accordingly, the attainable level of immunocompetence tends to decline as throughput increases. Level 0 models can be readily deployed in multiwell barrier formats for screening. Level 1 systems remain compatible with moderate-throughput studies of acute inflammation, chemotaxis, and transendothelial migration, and this is the operational space in which much of the field currently resides [109]. By contrast, Level 2 platforms generally move into low-throughput, custom-built configurations because antigen-specific T-cell responses require coordinated control over cell source, APC function, spatial organization, and culture duration [28]. Level 3 remains aspirational and, if realized, will almost certainly operate at proof-of-concept scale. This gradient is already visible in current practice, with primary-cell breathing alveolus systems occupying an intermediate position between highly parallelized screening formats and bespoke immune-enabled devices [108].

The key design principle is that the throughput-biomimicry operating point should be consciously chosen based on the stage of research. Early-stage discovery benefits from higher throughput and lower fidelity; mechanistic validation requires higher fidelity at the expense of throughput; and clinical translation demands patient-specific fidelity with sufficient throughput to capture population heterogeneity.

In summary, the engineering principles discussed above can be synthesized into a unified decision framework. This framework reveals a fundamental asymmetry: the engineering requirements for innate immune functions (Level 1) are substantially more forgiving than those for adaptive immune functions (Levels 2–3). Whereas Level 1 can be achieved with off-the-shelf immune cell lines and standard PDMS devices, Level 2 demands autologous or HLA-matched cellular components, long-term culture stability exceeding 14 days, and spatially organized antigen-presentation niches. These more stringent requirements largely account for why the field remains predominantly at Level 1. Throughout the following disease application sections, we apply this framework to critically assess the immunocompetence level attained by each platform.

### 3.6. Current State of Level 2 Immunocompetent LOC Systems

Level 2 LOC systems represent an important intermediate stage in which immune integration extends beyond innate inflammatory responses toward adaptive immune functionality. Such platforms may reproduce processes including leukocyte recruitment, macrophage activation, neutrophil migration, T-cell cytotoxicity [105], and, in more advanced cases, antigen-specific T-cell responses [28,106]. However, these models generally remain below Level 3 because they do not yet reproduce the broader coordination of adaptive immunity across spatially and temporally connected immune compartments. Key missing features include sustained antigen presentation, clonal expansion, B-cell responses, lymphoid organization, memory formation, and long-term immune homeostasis. Thus, the transition from Level 2 to Level 3 is defined not simply by adding additional immune cell types, but by establishing coordinated, antigen-specific, and dynamically regulated immune networks that extend beyond the local lung compartment.

## 4. Applications in Pulmonary Disease Modeling and Immunotherapy Evaluation

The value of immune integration depends on the biological question: epithelial-only systems cannot capture immune-mediated clearance in infection, macrophage crosstalk with fibroblasts is essential for TGF-β-driven fibrosis, and T cells are required for checkpoint blockade assessment. These context-specific limitations motivate immune integration to Levels 1–3 according to the intended application. As lung-on-a-chip (LOC) technology has progressed from structural biomimicry to functional immune modeling, its value has shifted accordingly. These systems are no longer used only to reproduce barrier physiology, but increasingly to interrogate how pulmonary immune responses are initiated, amplified, and therapeutically modulated [110] (Figure 5a). In this section, we examine respiratory infection, chronic inflammation and fibrosis, and immunotherapy evaluation as three major application domains through the lens of the immunocompetence framework introduced above. For each platform, we assess the level of immune competence actually achieved and identify the engineering features directly responsible for the biological insights obtained, rather than accepting immunological relevance as an implicit claim. This distinction is important because many studies are described as immunocompetent while remaining functionally restricted to Level 0 or Level 1.

### 4.1. Modeling Respiratory Infections

Respiratory infection is the most mature application area for immunocompetent LOCs. By combining an air–liquid interface, epithelial–endothelial compartmentalization, perfusion, and selected innate immune populations, these platforms can capture early host–pathogen dynamics with a temporal and spatial resolution not readily achievable in conventional static systems. At present, however, the field remains dominated by Level 1 models, meaning that acute innate responses can be resolved in detail, whereas adaptive immune clearance, memory formation, and humoral responses remain largely inaccessible.

#### 4.1.1. Viral Kinetics and Microenvironmental Modulation

One major advantage of infection chips is their ability to resolve the kinetics of early antiviral defense. Man et al. leveraged an influenza-infected LOC in conjunction with Luminex multiplex assays to track immune responses at an hourly resolution. This approach enabled the precise observation of macrophage phagocytosis, inflammatory cytokine release, and cell-death dynamics, demonstrating the platform’s ability to resolve the temporal sequence of host defense mechanisms. This study achieved immunocompetence Level 1 and illustrates how the integration of macrophages with a viral challenge in a mechanically active LOC can reveal therapeutic targets invisible in macrophage-free systems. Beyond acute infection, an LOC can elucidate how pre-existing conditions alter viral susceptibility. Nawroth et al. established an asthmatic model infected with human rhinovirus 16 (HRV16), revealing that an IL-13-induced Th2 microenvironment exacerbates inflammation by driving excessive epithelial cytokine secretion (IL-6, CXCL10) and promoting goblet cell hyperplasia. Crucially, this model identified that the CXCR2 antagonist MK-7123 could inhibit the resulting neutrophil transepithelial migration, highlighting the potential for targeted therapeutic intervention [111]. These studies demonstrate that even Level 1 immunocompetence can yield clinically actionable insights.

#### 4.1.2. Bacterial Pathogenesis and Immune Cell Migration

An LOC is particularly valuable in modeling the complex recruitment of circulating immune cells across the endothelial barrier during bacterial infections. Thacker et al. reconstructed the early stages of Mycobacterium tuberculosis (Mtb) infection, capturing the spatiotemporal dynamics of macrophage and neutrophil chemotaxis towards the infection site (Figure 5b) [112]. The platform achieved Level 1 immunocompetence by co-culturing epithelial cells, endothelial cells, and macrophages, and by enabling real-time imaging of mycobacterial colonization at the epithelial and endothelial interfaces. Mahieu et al. systematically benchmarked LOCs against transwell and organoid models for bacterial pneumonia modeling, demonstrating that LOCs uniquely capture the endothelial barrier component of immune cell transmigration, a process that is entirely absent in epithelium-only models [113]. All platforms evaluated in that comparison operated at Level 1 or below, as none incorporated adaptive immune components. Similarly, regarding opportunistic pathogens, Marhuenda et al. further extended this capability by incorporating ECM hydrogels into an acute respiratory distress syndrome (ARDS)-on-a-chip model, showing that matrix composition directly modulates neutrophil migration velocity and directionality during bacterial challenge [114]. The model achieved Level 1 through its demonstration of stimulus-induced chemotaxis and cytokine cascades.

#### 4.1.3. Fungal Invasion and Structural Pathology

The versatility of an LOC extends to invasive fungal diseases, which involve complex physical interactions between hyphae and host tissues. Barkal et al. developed a microscale human bronchial chip to simulate invasive pulmonary aspergillosis. By inoculating Aspergillus fumigatus spores directly onto the epithelial interface, the model successfully recapitulated key pathological events, including hyphal penetration of the epithelial barrier and the subsequent induction of IL-8-mediated neutrophil migration (Figure 5c) [115]. This study validated the unique capability of an LOC to model the structural pathology of fungal infections, offering a superior alternative to animal models for studying pathogen-specific immune responses. The model achieved Level 1 immunocompetence through the inclusion of macrophages in the vascular compartment, enabling observation of transendothelial migration in response to fungus-derived chemotactic gradients.

#### 4.1.4. The Lung Microbiome

Beyond defined pathogens, the resident pulmonary microbiome is increasingly recognized as a key modulator of baseline immune tone and barrier function. Commensal bacteria such as Streptococcus, Veillonella, and Prevotella influence alveolar macrophage polarization, epithelial antimicrobial peptide secretion, and neutrophil recruitment [116]. Mechanistically, lung microbiota-derived short-chain fatty acids (SCFAs) coordinate alveolar macrophage and γδT cell activation, revealing a direct link between microbial metabolites and innate immune surveillance [117]. However, conventional in vitro models fail to replicate the complex host–commensal interface under physiological conditions of flow, mechanical strain, and immune compartmentalization that are uniquely recapitulated in LOC platforms. Progress toward integrating the microbiome into LOC platforms is already underway. A recent review highlighted OOC and organoid technologies as promising platforms for replicating host–microbiome interactions in the lung, with existing studies primarily focused on modeling pathogen invasion—such as Mycobacterium tuberculosis and influenza A virus—rather than commensal-mediated immune regulation [118]. Although host–microbe interactions have been investigated in selected airway/lung microphysiological models, we are not aware of a platform that simultaneously integrates a defined commensal microbial community with functionally validated innate and adaptive immune compartments, representing a significant gap between current on-chip models and physiological mucosal immunity. A near-term priority should be the incorporation of simplified synthetic microbial consortia or patient-derived respiratory microbiomes into the airway compartment of existing Level 1 platforms, enabling controlled investigation of how commensal-derived metabolites (e.g., SCFAs) modulate alveolar macrophage polarization, neutrophil recruitment, and epithelial barrier function under breathing-like stretch and fluid flow. This integration would also allow mechanistic dissection of how microbial metabolites regulate pulmonary immune homeostasis that remains largely unexplored in current LOC systems and whose elucidation will be essential for progressing toward Level 2–3 immunocompetence.

**Figure 5:**
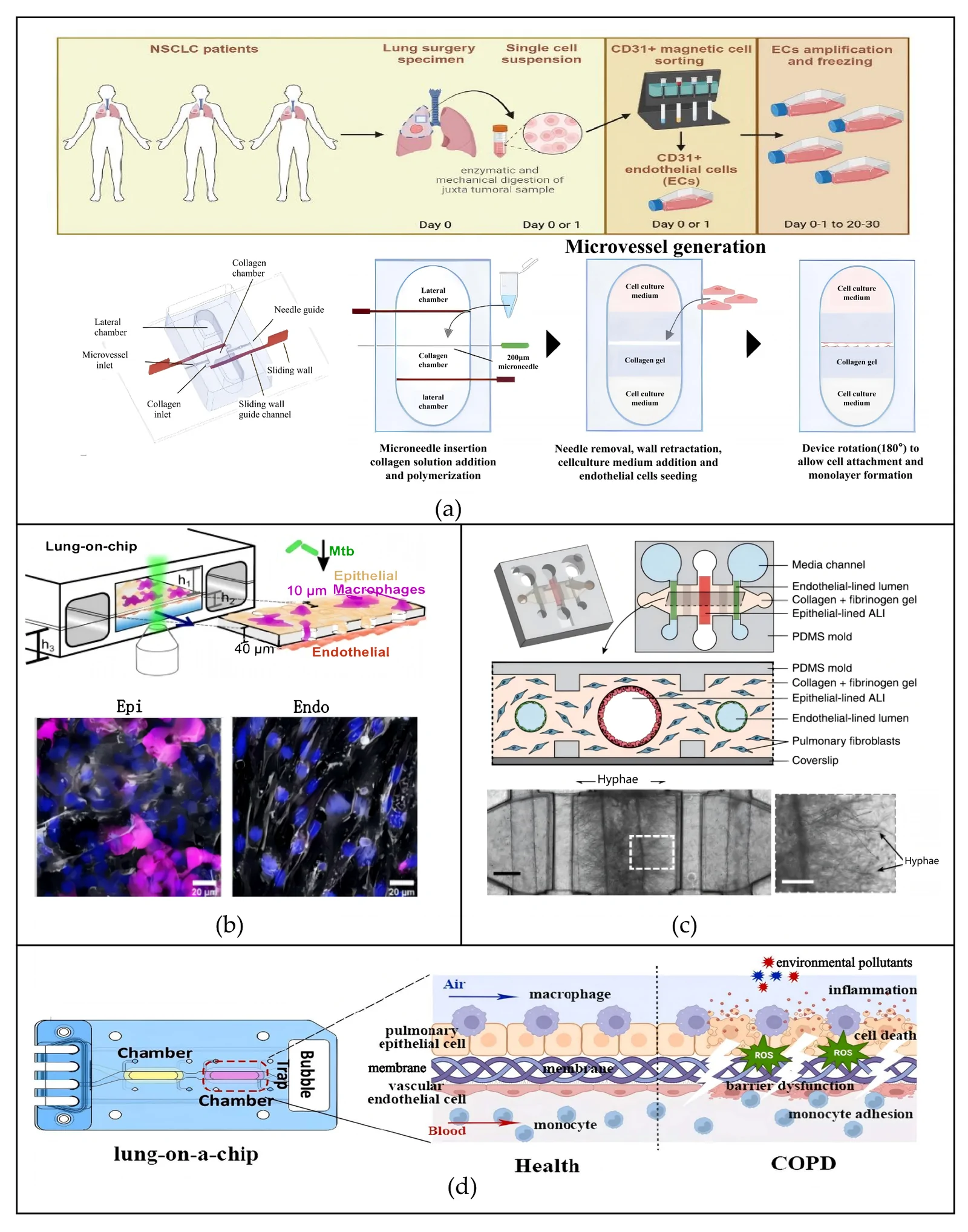
Applications of lung-on-a-chip technology in pulmonary immune disease modeling. (**a**) Patient-derived lung endothelial cell isolation and transcriptomic characterization. Overview of isolation of endothelial cells (ECs) from tumor tissue of surgical NSCLC samples, the PDMS microfluidic chip fabrication, and microvessel generation. Ref. [110]. (**b**) Microfluidic model of Mycobacterium tuberculosis infection, showing colonization of epithelial and endothelial compartments in a co-culture containing alveolar epithelial cells, macrophages, and endothelial cells. Ref. [112]. (**c**) Alveolar-capillary infection model of invasive pulmonary aspergillosis, in which A. fumigatus spores reproduce fungal invasion, hyphal penetration of the epithelial barrier, and inflammatory activation. Ref. [115]. (**d**) Inflammatory lung disease model integrating resident macrophages and circulating monocytes to reproduce immune surveillance, cytokine amplification, monocyte adhesion, reactive oxygen species (ROS) production, and barrier injury under cigarette smoke exposure. Ref. [119].

Respiratory infection chips have established Level 1 immunocompetence as the current operational standard. Their strongest contributions are those that depend specifically on integrated architecture and live spatiotemporal readout: real-time visualization of pathogen entry and barrier breach, direct observation of neutrophil and monocyte trafficking under flow, and demonstration that epithelial and mechanical context shape the magnitude and trajectory of innate defense [21,112]. These are genuine LOC-enabled discoveries rather than miniaturized reproductions of known biology. By contrast, several important aspects of infection immunity remain largely outside current chip capability. These include antigen-specific T-cell responses, long-term pathogen clearance, immunological memory, and class-switched humoral immunity. As a result, many questions central to vaccine biology and durable host protection still depend on animal models, clinical studies, or reductionist ex vivo systems for validation. The major barrier is not simply the absence of lymphocytes, but the combined constraint of short experimental windows, lack of pathogen-specific naïve T and B cells, and the unresolved adaptive immune bottleneck. For this reason, most current infection chips should be interpreted as high-resolution models of early innate immune dynamics rather than comprehensive models of respiratory immunity. Extending this perspective, the role of the resident pulmonary microbiome—as opposed to defined pathogens—in shaping the baseline immune tone and modulating host susceptibility remains almost entirely unexplored in current LOC platforms.

### 4.2. Modeling Chronic Inflammation and Pulmonary Fibrosis

Beyond acute infections, LOC models the progressive pathology of chronic inflammatory diseases, bridging the gap between simple cell cultures and complex in vivo responses. However, the fibrosis literature in particular has suffered from a conflation of LOC-derived evidence with findings from animal models and conventional culture systems. In addition to fibrosis, LOC platforms have been applied to study inhalation toxicology and environmental exposures, including particulate matter and nanomaterial-induced pulmonary injury [119].

In COPD research, Yang et al. engineered an LOC with integrated macrophage and monocyte surveillance in which cigarette smoke extract triggered coordinated release of IL-6, TNF-α, and MCP-1. The model identified IL-17A as a central driver of neutrophilic inflammation, achieving Level 1 immunocompetence [119] (Figure 5d). For asthma, Nawroth et al. integrated mast cells into a breathing airway-on-chip and demonstrated that IL-13 alone was sufficient to increase neutrophil recruitment during rhinovirus infection, but that mast cell-derived mediators were required for the full asthma-like phenotype, including goblet cell metaplasia. Cyclic stretch directly enhanced IL-13-induced mucus production, establishing a causal link between mechanical actuation and a disease-relevant output. The model remained at Level 1, as it captured innate and mechanically driven components of asthma pathology without antigen-specific adaptive responses [111]. Du et al. subsequently identified elevated CXCL14 in sputum from asthma patients and correlated this with eosinophilic inflammation [120]. A distinct Th2-driven immune axis is absent from the model. This discrepancy between on-chip models and clinical observations illustrates a broader principle: eosinophilic, adaptive immune-driven disease features lie beyond the reach of current Level 1 platforms, and clinical biomarker studies provide externally validated benchmarks against which higher-level LOC platforms should ultimately be measured.

For pulmonary fibrosis, chip models co-culturing alveolar epithelial cells and fibroblasts have elucidated the dynamic relationship between TGF-β signaling and macrophage polarization. Mejías et al. demonstrated in a 96-well-format LOC that TGF-β stimulation of epithelial–endothelial co-cultures induced fibroblast activation and collagen deposition in a macrophage-dependent manner, and the high-throughput format enabled dose–response profiling of Nintedanib and pirfenidone across multiple donors. The model achieved Level 1 immunocompetence [109]. More recently, Xia et al. developed a micro-lung chip in which nanoparticle-mediated delivery of a TGF-β receptor inhibitor specifically to macrophages suppressed collagen I deposition and α-SMA expression while sparing epithelial cells [121]. This spatial selectivity was directly enabled by the compartmentalized LOC architecture and represents a level of mechanistic resolution inaccessible in animal models. Several fibrosis-relevant pathways, however, remain characterized only in animal systems and await LOC validation: PI3K-AKT signaling in myofibroblast differentiation, demonstrated in the rat bleomycin model [122]. Similarly, bone marrow-derived MSC transplantation shows anti-fibrotic efficacy across multiple animal models [123] potentially through paracrine modulation of macrophage polarization [124], but whether these effects persist within the biophysically distinct microenvironment of a breathing LOC remains untested. We identify these as high-priority validation targets, recognizing that reductionist findings cannot be assumed to translate to the chip environment without explicit experimental confirmation.

LOC-based fibrosis studies have established that macrophages function not merely as effector cells but as spatial orchestrators whose positioning within the epithelial–endothelial architecture determines fibrotic outcome, an insight inaccessible to macrophage-knockout animals or simple co-cultures. We organize the fibrosis evidence base into three tiers. LOC-direct discoveries: macrophage spatial positioning as a determinant of fibrotic versus resolving outcomes; TGF-β-mediated epithelial–mesenchymal crosstalk visualized in real time on-chip; and the demonstration that cyclic mechanical strain modulates fibroblast activation independently of biochemical stimuli. LOC-supported findings: the pro-fibrotic role of M2-polarized macrophages and the anti-fibrotic potential of macrophage reprogramming strategies, both corroborating animal model observations. High-priority targets awaiting LOC validation: PI3K-AKT-driven myofibroblast differentiation; MSC-derived exosome mechanisms; and the contributions of Th2 and Th17 CD4^+^ T cells, strongly implicated in human IPF yet entirely unexplored in chip-based models. The principal limitation is that current fibrosis models remain at Level 1; adaptive immune cells define the critical gap for Level 2 advancement.

### 4.3. Preclinical Evaluation of Immunotherapies and Vaccines

The distinct advantage of an LOC in immunotherapy evaluation lies in its ability to model human-specific immune responses, including toxicities that animal models fail to predict. Unlike infection and fibrosis models, immunotherapy evaluation typically demands Level 2 immunocompetence: the platform must support antigen-specific T cell activation, target cell killing, and cytokine-mediated crosstalk. We note, however, that the tumor immune microenvironment involves far more than the CD8^+^ T cell–PD-L1 axis. A comprehensive tumor-on-chip should also account for tumor-associated macrophages (TAMs), myeloid-derived suppressor cells (MDSCs), neutrophils, and metabolic suppressive mechanisms such as adenosine accumulation and hypoxia-driven immune exclusion. Cancer-associated fibroblasts further contribute through ECM deposition and chemokine sequestration. Current LOC tumor models have begun addressing stromal contributions to drug resistance [31] and endothelial checkpoint-mediated T cell suppression [110], but the systematic integration of the full myeloid suppressor axis remains an open challenge.

#### 4.3.1. Tumor Immunotherapy and Efficacy Evaluation

An LOC provides a physiologically relevant platform to dissect the tumor immune microenvironment. Tan et al. demonstrated in an NSCLC model that tumor-associated stromal cells significantly promoted resistance to EGFR-TKIs through paracrine HGF secretion, an effect that was absent in tumor-cell-only cultures [31]. This finding illustrates the principle that stromal context, specifically, the presence of cancer-associated fibroblasts, directly modulates drug response, a discovery enabled by the multi-compartment LOC architecture (Level 0–1). Lansche et al. developed a patient-derived vascularized lung tumor-on-chip (Figure 6a) and demonstrated that tumor-associated endothelial cells actively suppress CD8^+^ T cell effector function through upregulation of PD-L1 and Galectin-9 [110]. Jung et al. and Veith et al. further advanced scalability and patient specificity: Jung achieved uniform lung cancer organoid formation and perfusion for high-throughput drug testing [125], while Veith maintained autologous T lymphocytes within a tumor-on-chip to quantify tumor killing within 24 h [106]. Platforms demonstrating antigen-specific T-cell responses can be classified as Level 2 immunocompetent models; however, the absence of organized lymphoid structures and coordinated adaptive immune maturation currently precludes Level 3 classification [125] (Figure 6b).

#### 4.3.2. Immune-Related Adverse Events

LOCs have proven particularly valuable for predicting human-specific immunotoxicity undetected by animal models. Kerns et al. used a humanized lung chip to evaluate a T cell-bispecific antibody targeting FOLR1 and accurately predicted severe pulmonary toxicity, T cell infiltration, epithelial apoptosis, and pro-inflammatory cytokine release that later manifested as grade 3–4 pneumonitis in Phase I trials, despite normal lung histology in cynomolgus monkeys. The Level 2 LOC succeeded where animal models failed because it incorporated human primary cells expressing physiological FOLR1 levels [105] (Figure 6c). LOCs have also been applied to model cytokine release syndrome (CRS) in CAR-T therapy, wherein engineered T-cell activation triggers a barrier-disrupting cytokine storm. Current platforms, however, recapitulate only the early acute phase of CRS; modeling the full clinical spectrum from mild fever to multi-organ failure will require integrated multi-organ chips with extended culture durations and real-time hemodynamic monitoring.

#### 4.3.3. Vaccine Development and Mucosal Immunity

LOCs offer a promising platform for studying pulmonary mucosal immunity, which depends on IgA transcytosis, tissue-resident memory cells, and respiratory tract-specific antigen-presenting cell networks. We emphasize that vaccine evaluation in LOC platforms is an emerging opportunity rather than an established application: most immunological outcomes in this area have been demonstrated in animal models or conventional ALI cultures, with direct LOC validation remaining sparse. Airway inoculation in LOC models can induce mucosal IgA responses [107,109], and for tuberculosis, preclinical studies suggest that mucosal delivery of viral-vectored vaccines induces superior lung-resident memory T-cell responses compared with parenteral immunization [108]. In safety assessment, a candidate RSV vaccine tested on-chip successfully predicted vaccine-associated enhanced respiratory disease (VAERD) subsequently observed in clinical trials [107]. However, all current vaccine-related LOC models remain at Level 1 or below: none have demonstrated antigen-specific B cell activation, class-switched antibody secretion, or quantitative comparison of mucosal IgA versus systemic IgG responses within the chip. Closing these three specific capability gaps is the prerequisite for a vaccine-evaluation LOC to achieve Level 2 immunocompetence and establish credibility for regulatory applications.

Overall, chronic inflammation and fibrosis models remain predominantly at Level 1 immunocompetence. Their major strengths lie in resolving prolonged innate and stromal crosstalk under realistic mechanical and architectural constraints, rather than in reconstructing the adaptive immune circuits that sustain chronic disease in vivo. This distinction matters because many clinically important questions in fibrosis, not least the roles of Th2, Th17, and tissue-resident lymphocyte populations, are unlikely to be addressed credibly without progress toward Level 2. Until such systems are achieved, mechanistic claims about adaptive immune drivers of chronic lung disease should be treated as extrapolations unless directly demonstrated on-chip.

**Figure 6:**
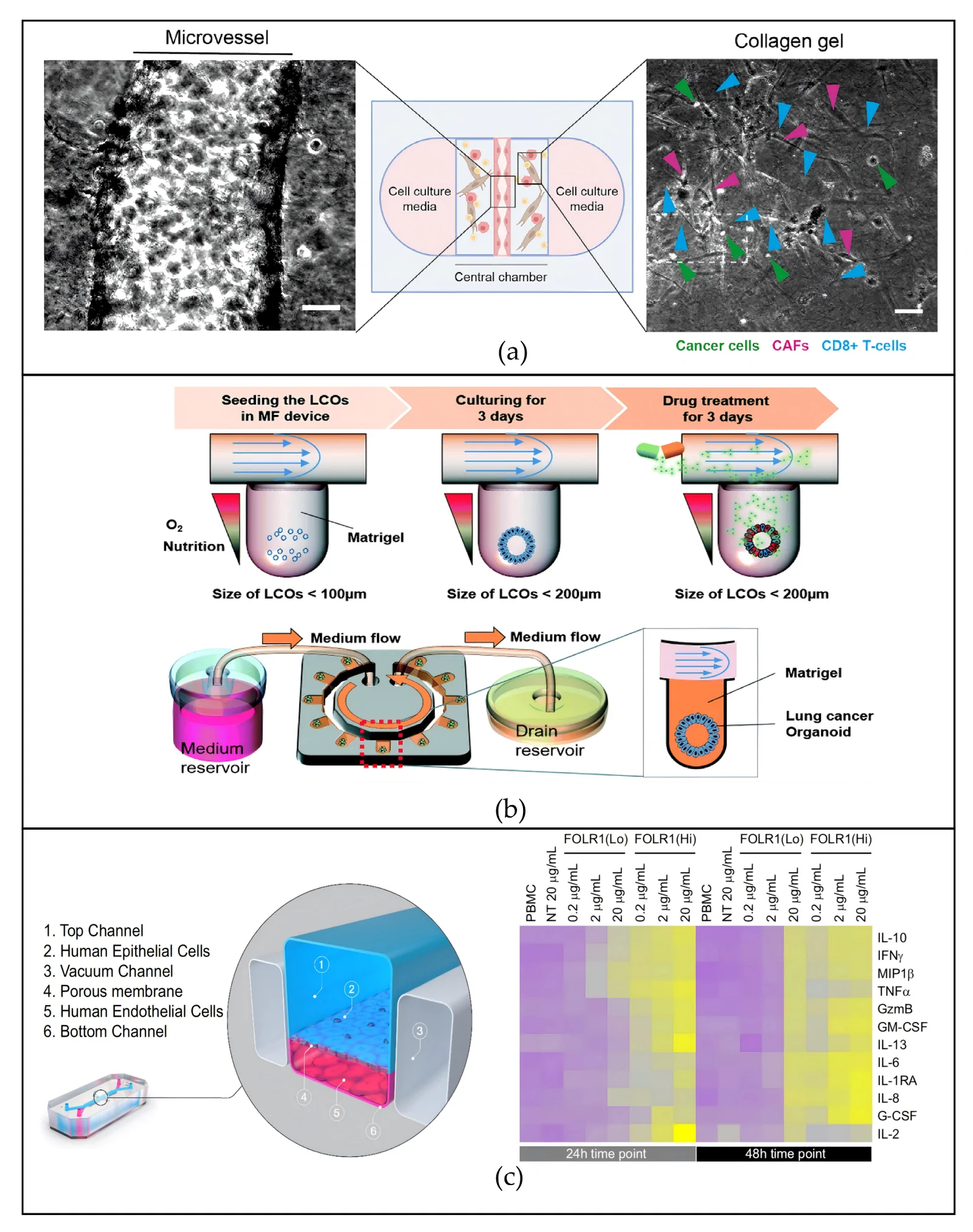
Applications of LOCs in immunotherapy evaluation and drug screening. (**a**) Lung tumor chip showing CD8^+^ T cells, autologous lung cancer cells, and fibroblasts, depicting T cell infiltration into the tumor region, allowing assessment of T cell anti-tumor activity and immunotherapy response. Ref. [110]. (**b**) Patient-derived lung cancer organoids applied in microfluidic chips for drug sensitivity testing, featuring uniform culture under flow and drug response detection, supporting high-throughput immunotherapeutic drug screening and target research. Ref. [125]. (**c**) Humanized LOC for predicting pulmonary toxicity of FOLR1-TCB. High-affinity FOLR1-TCB induced T cell activation, PBMC attachment, epithelial apoptosis, and significant pro-inflammatory cytokine release, predicting TCB-related immune adverse events. Ref. [105].

## 5. Summary and Prospects

Lung-on-a-chip (LOC) technology has evolved from a platform for structural biomimicry into a promising framework for modeling human pulmonary immunity. Its value is already evident in patient-derived disease modeling, immuno-oncology safety assessment, and drug screening, including the evaluation of complex natural products and traditional Chinese medicine formulations. However, as emphasized throughout this Review, progress in immunological realism has been uneven. Most current pulmonary LOCs remain at Level 1 immunocompetence, only a small minority approach Level 2, and Level 3 lymphoid organization has not yet been achieved.

Three coupled barriers now define the field. First, temporal stability remains limited: most immune-enabled chips sustain functional co-culture for only 3–8 days [107], whereas clinically relevant adaptive immune responses typically require 14–21 days. Second, standardization is still immature. Variation in cell source, chip material, perfusion settings, and immune readouts reduces reproducibility across studies [126], the throughput biomimicry trade-off constrains scalability [127] and PDMS adsorption can distort lipid handling and chemokine recovery, complicating quantitative immune interpretation [128]. Third, adaptive immune integration remains the principal engineering frontier [129]. Antigen-specific responses require coordinated antigen presentation, stable APC–T-cell contact, donor matching, and media compatibility with epithelial differentiation, requirements that current systems rarely satisfy simultaneously.

A realistic roadmap is therefore tiered. In the near term, the priority is to consolidate advanced Level 1 platforms through immune-specific quality metrics, benchmarked immune-cell sources, and non-destructive readouts of barrier function, cytokine dynamics, and metabolism, together with minimal reporting standards and standardized patient-sample workflows. In the midterm, progress toward Level 2 will depend on protected priming niches, modular media strategies, and interconnected lung–lymph node platforms that support antigen-specific adaptive immunity, including T cell clonal expansion, effector cytokine production, and class-switched antibody responses. In the longer term, Level 3 ambitions, organized lymphoid structures with germinal center-like activity, will require durable biomaterials, spatially controlled immune architecture, and broader multi-organ integration.

Several cross-cutting priorities will shape this transition. First, increasingly complex immunocompetent LOCs will require more informative measurement systems, combining real-time sensing, live-cell imaging, and endpoint single-cell multi-omics, so that added complexity yields mechanistic insight rather than irreproducible black boxes. Second, artificial intelligence and machine learning will become essential for integrating these multidimensional datasets, enabling trajectory inference, causal network modeling, multimodal data fusion, and, ultimately, patient-specific digital twins for response prediction. This is particularly relevant as emerging high-sensitivity analytical technologies generate signals whose interpretation may depend on computational denoising and pattern recognition. Third, the biological scope of pulmonary LOCs must expand to include underexplored regulators such as immunometabolism, neuro–immune–epithelial crosstalk, and circadian immune rhythms. Finally, regulatory translation must advance in parallel through shared evidentiary standards and tiered testing pipelines aligned with preclinical decision-making.

In summary, LOCs should become more rigorously functional. Future progress depends on three shifts: from cell inventory to functional benchmarking, from isolated tissue models to connected immune axes, and from descriptive observation to multimodal, computationally assisted interpretation. If these shifts can be realized, immunocompetent pulmonary LOCs may progress from sophisticated microphysiological models to genuinely predictive platforms for disease modeling, immunotherapy evaluation, and reduced reliance on animal testing in drug development.

## Figures and Tables

**Figure 1:**
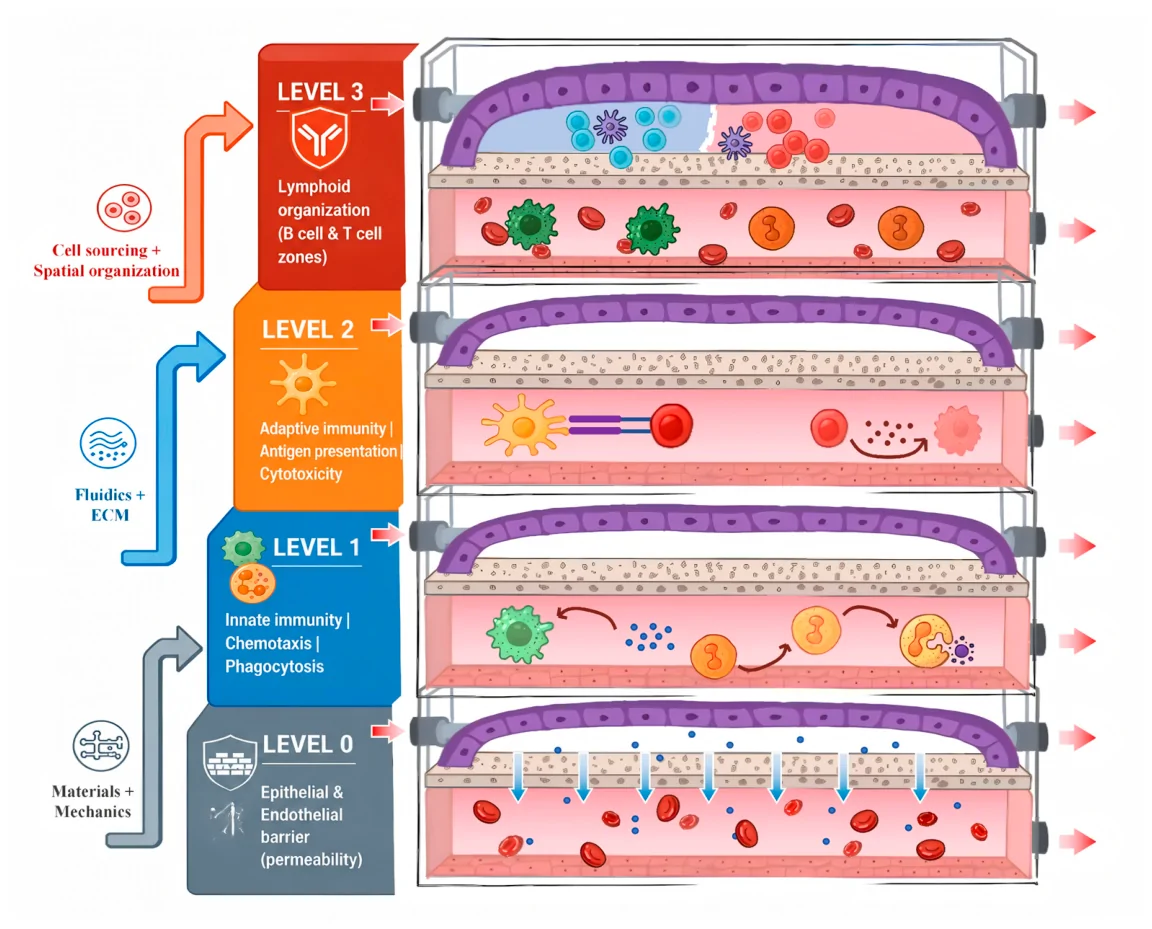
A functional grading framework for immunocompetent lung-on-a-chip. The framework defines four escalating levels of immune function: Level 0 captures basic barrier integrity and permeability; Level 1 introduces innate immune activities including chemotaxis and phagocytosis; Level 2 achieves adaptive immunity through antigen presentation and T-cell activation; and Level 3 reconstitutes organized lymphoid structures with segregated B-cell and T-cell zones.

**Figure 2:**
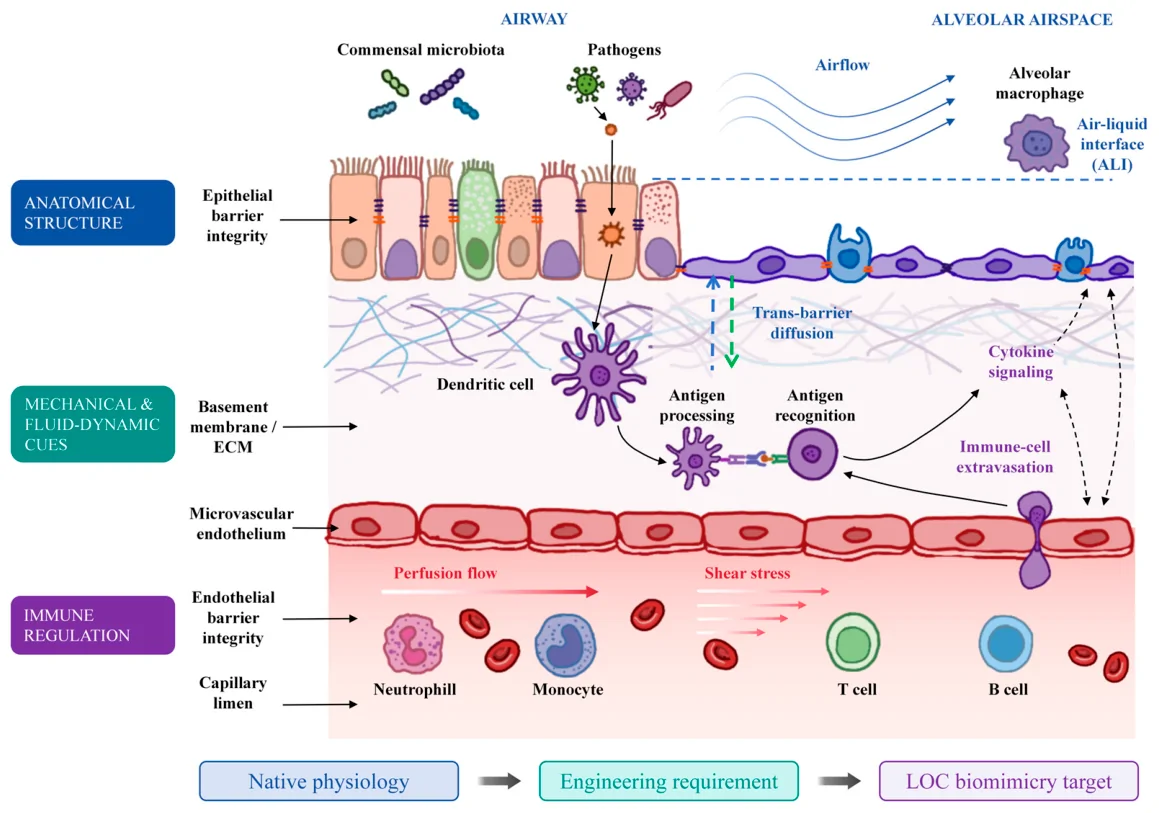
Physiological principles guiding the design of an immunocompetent lung-on-a-chip model.

**Table 1:** Pulmonary model architectures and their immunocompetence capabilities.

Capability Dimension	Transwell/ALI	Organoid	Precision-Cut Lung Slice	LOC	Hybrid Model
Barrier and innate immunity	✓✓✓	✓✓	✓✓✓	✓✓✓	✓✓✓
Immune cell trafficking	✗	✗	✓	✓✓✓	✓✓✓
Adaptive immune responses	✗	✗	✗	✓✓	✓✓✓
Fibrosis and stromal crosstalk	✗	✓	✓✓✓	✓✓	✓✓✓
Throughput and scalability	✓✓✓	✓✓	✗	✓✓	✓

Symbols: ✓✓✓ = Well-established/robustly capable; ✓✓ = Moderate capability; ✓ = Limited but possible; ✗ = Not feasible.

**Table 2:** Classification rubric.

Level	Immune Inclusion	Spatial Organization	Functional Validation	Adaptive Immunity
0	None/minimal	None	None	No
1	Immune cells included	Basic compartmentalization	General inflammatory response	Not required
2	Relevant immune populations	Physiologically meaningful	Dynamic/functional immune response	May include antigen-specific response
3	Multicellular immune ecosystem	Tissue-like organization	Coordinated innate-adaptive immunity	Adaptive maturation/memory/lymphoid organization

## Data Availability

No new data were created or analyzed in this study. Data sharing is not applicable to this article.

## Abbreviations

The following abbreviations are used in this manuscript:

ACI	Alveolar–capillary interface
ALI	Air–liquid interface
AM	Alveolar macrophage
ARDS	Acute respiratory distress syndrome
BMSC	Bone marrow-derived mesenchymal stem cell
CAR-T	Chimeric antigen receptor T-cell
CCR	C-C chemokine receptor
COPD	Chronic obstructive pulmonary disease
CRS	Cytokine release syndrome
DC	Dendritic cell
ECM	Extracellular matrix
EGFR-TKI	Epidermal growth factor receptor tyrosine kinase inhibitor
FOLR1	Folate receptor 1
FSS	Fluid shear stress
GLP	Good Laboratory Practice
GM-CSF	Granulocyte-macrophage colony-stimulating factor
HLA	Human leukocyte antigen
ICAM-1	Intercellular adhesion molecule 1
ICI	Immune checkpoint inhibitor
IFN	Interferon
IL	Interleukin
ILC2	Type 2 innate lymphoid cell
IMC	Imaging mass cytometry
IPF	Idiopathic pulmonary fibrosis
iPSC	Induced pluripotent stem cell
irAE	Immune-related adverse event
LOC	Lung-on-a-chip
LPS	Lipopolysaccharide
MCP-1	Monocyte chemoattractant protein 1
MHC	Major histocompatibility complex
MMP9	Matrix metalloproteinase 9
MSC	Mesenchymal stem/stromal cell
NK	Natural killer
NLRP3	NLR family pyrin domain containing 3
NSCLC	Non-small cell lung cancer
PBMC	Peripheral blood mononuclear cell
PD-L1	Programmed death-ligand 1
PDMS	Polydimethylsiloxane
PI3K	Phosphoinositide 3-kinase
PPARγ	Peroxisome proliferator-activated receptor gamma
scRNA-seq	Single-cell RNA sequencing
SP-A/SP-D	Surfactant protein A/D
TCB	T-cell bispecific antibody
TGF-β	Transforming growth factor beta
Th	T helper
TIME	Tumor immune microenvironment
TLR	Toll-like receptor
TLS	Tertiary lymphoid structure
TNF-α	Tumor necrosis factor alpha
T_RM	Tissue-resident memory T cell
TRPV4	Transient receptor potential vanilloid 4
VCAM-1	Vascular cell adhesion molecule 1
YAP/TAZ	Yes-associated protein/transcriptional co-activator with PDZ-binding motif
